# The effect of *Beauveria bassiana* inoculation on plant growth, volatile constituents, and tick (*Rhipicephalus appendiculatus*) repellency of acetone extracts of *Tulbaghia violacea*

**DOI:** 10.14202/vetworld.2020.1159-1166

**Published:** 2020-06-20

**Authors:** Pumla Staffa, Nkululeko Nyangiwe, George Msalya, Yakob Petro Nagagi, Felix Nchu

**Affiliations:** 1 Department of Horticultural Sciences, Cape Peninsula University of Technology, Bellville, Symphony Way, Bellville, Cape Town, 7535, South Africa; 2 Department of Agriculture, Dohne Agricultural Development Institute, Private Bag X15, Stutterheim, 4930, South Africa; 3 Department of Animal, Aquaculture and Range Sciences, Sokoine University of Agriculture, P. O. Box 3004, Chuo Kikuu, Morogoro, Tanzania; 4 Livestock and Human Diseases Vector Control Division, Tropical Pesticides Research Institute, P. O. Box 3024, Arusha, Tanzania

**Keywords:** *Beauveria bassiana*, fungal endophyte, secondary metabolites, tick repellency, tick toxicity, *Tulbaghia violacea*

## Abstract

**Aim:**

The purpose of the present study was to evaluate the effects of *Beauveria bassiana* (Hypocreales) inoculum on plant growth, volatile constituents, and tick repellency of the extracts of *Tulbaghia violacea* (Amaryllidaceae).

**Materials and Methods:**

Eight-week-old potted seedlings of *T. violacea* were each inoculated with conidia of *B. bassiana* (strain SM3) suspended at a concentration of 1×10^6^ conidia mL^−1^. Tissue colonization by fungal conidia was assessed after 3 weeks. Plant growth, volatile constituents, and tick repellency were assessed after 12 weeks post-treatment.

**Results:**

*B. bassiana* conidia successfully colonized leaf and root tissues of *T. violacea*. The growth of fungal hyphae out of the leaf and root sections occurred in 75% and 91.6% of plants, respectively. Inoculation of the plants with *B. bassiana* significantly (p<0.05) influenced root length and plant height but did not have substantial effects on weights and leaf number of *T. violacea*. While the fungus did not have significant effects on overall number of the volatile chemical constituents, significant variations in the quantity (area ratio) were observed in at least four compounds that were detected. In the tick repellency bioassay, high concentration (20 w/v%) of acetone extract from fungus-exposed plants produced the least repellent effect on *Rhipicephalus appendiculatus* larvae (Ixodidae), while at lower concentrations (5 w/v% and 10 w/v%) of acetone extracts of *T. violacea*, tick repellent activity of the extract of the fungus treatment was significantly improved and was comparable to commercial N,N-Diethyl-m-toluamide and the other treatments.

**Conclusion:**

Experimental fungal inoculation positively influenced plant growth in height and root length and tick (*R. appendiculatus*) repellency of acetone extracts of *T. violacea* at a concentration of 10 w/v% compared to the control treatment.

## Introduction

Ticks are associated with many diseases of humans, livestock, and wildlife. Developing countries, especially those in Africa, are heavily burdened by tick infestations and record huge annual economic losses [1]. Ticks and tick-borne diseases (TBDs) have adverse impacts on livestock and human health, hence, placing a huge burden on the livelihoods of resource-poor farming communities in developing countries [2,3]. TBDs are among the most important diseases of livestock [4]. It is estimated that a TBD such as East Coast Fever (ECF) kills 1.1 million cattle in Africa resulting in economic losses of approximately $160 million annually [5]. The hard-bodied ticks are important vectors of pathogens; they are capable of transmitting a wide range of pathogens including bacteria, viruses, and parasites [6]. One of the most important tick species in the African continent is *Rhipicephalus* *appendiculatus*, the main vector of the protozoan pathogen *Theileria parva*, a causative agent of ECF in East Africa, corridor disease in Eastern and Southern Africa, and January disease in Central Africa [7]. ECF is associated with high cattle mortality [8]. There are fears that climate, land use, and vegetation changes will extend the distribution of *R. appendiculatus* in South Africa, resulting in increased incidences of *T. parva* outbreaks among cattle [9]. Synthetic chemical acaricides are widely used for controlling ticks, but there are worries over acaricide resistance and environmental contamination [10]. These concerns have favored the search for plant-based anti-tick agents, which are thought to be more sustainable and environmentally friendly than synthetic acaricides [11]. Traditional remedies including the use of ethnoveterinary plants are still the main approaches for the treatment of animal health problems, such as wounds, tick infestations, and TBDs in Africa, especially in resource-poor regions [12-14]. Ethnomedicinal knowledge has played a crucial role in the drug discovery and development processes [15,16]. Some herbal-based acaricides are available commercially [17,18]. It is worth mentioning that plant secondary metabolites are responsible for the bioactivities and medicinal properties of plants; hence, many studies are investigating ways to improve the medicinal constituents in plants [19,20]. Production of secondary metabolites in plants can be influenced by manipulating biotic and abiotic environmental factors [20,21]. Inoculation of plants with an endophytic fungus is a biotic approach that can be used to enhance the production of secondary metabolite in host plant species [22,23]. Fungal endophytes can colonize plant tissues and form symbiotic and mutual beneficial association with host plants [24]. Interestingly, some endophytic fungi may have detrimental effects on arthropod herbivores [25]. A fungal endophyte may protect plants from herbivory and disease [26], which is often mediated by changes in volatile and alkaloid constituents of host plants [27]. When a fungal endophyte colonizes plant tissues, it influences plant growth and secondary metabolite production [28-30].

The use of ethnoveterinary plants for tick control is widespread in Africa. Thus far, however, few plant species that are used traditionally for control of ticks have been scientifically validated [31]. Some species belonging to the Amaryllidaceae family, such as *Allium cepa* and *Allium sativum*, have acaricidal and tick repellent properties [32,33]. The genus *Allium* is well-recognized for their anti-tick activities and their bioactive secondary metabolites, including allicin (diallyl thiosulfinate) [34]. The genus *Tulbaghia* (Amaryllidaceae), which is closely related to the genus *Allium*, could be a potential source of tick control agents or extracts [35]. Crushed leaves of *Tulbaghia violacea* are used to repel ticks and mosquitoes [36]; however, currently, scientific reports on the efficacy of *Tulbaghia* on ticks are rare. In South Africa, *T. violacea* is also used traditionally for the treatment of many diseases, including pulmonary tuberculosis, intestinal worms, and sinus headaches [36]. *T. violacea* is frequently harvested from the wild by traditional healers, putting intense pressure on the wild populations [37,38]. Despite its medicinal uses, very few studies have focused on the optimization of the cultivation with the view of improving yield and quality of the medicinal materials, and bioactivity derived from this species [39].

In the current study, *T. violacea* was inoculated with an endophytic arthropod-pathogenic fungus with the purpose of increasing the secondary metabolite contents and anti-tick repellent activity of its extracts. Specifically, the effects of amending plant growth medium with the inoculum of *Beauveria bassiana* (Hypocreales) on plant growth, plant secondary metabolite, and *R. appendiculatus* repellent activity of extracts of *T. violacea* were evaluated.

## Materials and Methods

### Ethical approval

The Research Ethics Committee of the Faculty of Applied Sciences, Cape Peninsula University of Technology approved this study.

### Fungus

An indigenous strain (SM3) of *B. bassiana* was used in the present study. Cultures of the fungal strain were obtained from the Department of Horticultural Sciences, Cape Peninsula University of Technology (CPUT). Before its use, a conidial germination test to determine conidial viability was carried out according to the method described by Inglis *et al*. [40] with modifications. The viability of conidia was determined by spread plating 0.1 ml of conidia suspension, titrated at 1×10^6^ conidia ml^−1^ on half-strength Potato Dextrose Agar (PDA) plates amended with 0.02 g/L of ampicillin (Sigma-Aldrich, South Africa) and 0.04 g/L streptomycin (Sigma-Aldrich), and incubated at 26±2°C. Plates were then examined after 24 h by placing two sterile microscope cover slips on each plate and the percentage germination determined from 100-spore counts under each cover slip. The germination percentage was over 90%.

The fungus was cultured on half-strength PDA amended with 0.02 g/L of ampicillin (Sigma-Aldrich, Johannesburg, South Africa) and 0.04 g/L streptomycin (Sigma-Aldrich, Johannesburg, South Africa). Clean fungal subcultures on agar were prepared in 9 cm diameter Petri dishes and incubated at 25°C. *B. bassiana* conidia were harvested by scrapping 3-4-week-old cultures using sterile spatula and suspended into 100 ml bottles of sterile distilled water containing sterile 0.01% Tween 80. The suspension was mix using a vortex shaker for 5 min to ensure separation of spores. Conidial concentrations were determined using an improved Neubauer hemocytometer and the suspensions were adjusted to 1×10^6^ conidia mL^−1^ in sterile distilled water.

### Tick colonies

Nine-day-old adults of *R. appendiculatus* colony used in this study were obtained from the Division of Livestock and Human Diseases Vector Control of the Tropical Pesticides Research Institute (TPRI) in Arusha, Tanzania. The ticks were reared in a room that had relative humidity of 70% and a temperature range of 26-28°C. Ticks were kept in small tubes with gauze stopper. These small tubes are kept in small cylindrical containers that are half-filled with moist sand. Nymphs and larvae were fed on New Zealand white rabbit and adult ticks were fed on sheep. The mammals were handled humanely in accordance with ethical guidelines of the TPRI.

### Greenhouse experiment

**Figure-1:**
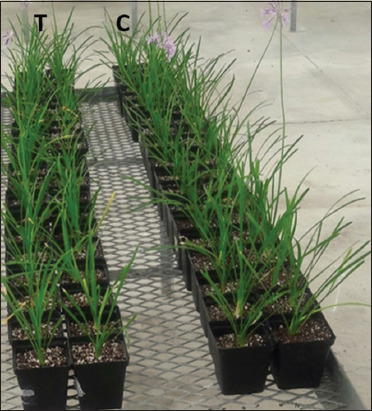
Fungus-treated potted *Tulbaghia violacea* group (T) and control-treated potted *T. violacea* (C).

The experiment was conducted in a greenhouse of the Department of Horticultural Sciences, CPUT, Bellville campus, Western Cape, Province, South Africa. Seedling trays of *T. violacea* were obtained from a wholesale nursery, Western Cape Seedlings, Cape Town. Eight-week-old seedlings were transplanted into 14 cm diameter pots containing substrate mix made of peat, silica sand, perlite, and vermiculite in equal volume and were placed in a controlled greenhouse. There were two treatments: Plants that were inoculated with the fungus *B. bassiana* (test group) and those that were not exposed to the fungus (control group) (Figure-1). Two hundred plants were randomly allocated to each block with 100 replicates per treatment. The potted plants were placed on flat surface steel tables (2.5×1 m). Plants were fed with Nutrifeed fertilizer obtained from Stodels Garden Centre in Cape Town and the fertilizer contained the following ingredients: 65 g/kg N, 27 g/kg P, 130 g/kg K, 70 mg/kg Ca, 20 mg/kg Cu, 1500 mg/kg Fe, 10 mg/kg Mo, 22 mg/kg Mg, 240 mg/kg Mn, 75 mg/kg S, 240 mg/kg B, and 240 mg/kg Zn. The nutrient solution was prepared by dissolving 60 g of the fertilizer in 60 L reservoir with tap water. Plants were irrigated once a week, each plant was watered with 100 mL distilled water containing Nutrifeed. *B. bassiana* conidial suspension (50 ml of 1×10^6^ conidia mL^−1^) was added each potted plant in the test treatments by drenching. Control treatment was not exposed to fungal spores, only 50 ml of sterile 0.01% Tween 80 was added to each control replicate. After 3 weeks, this treatment procedure was repeated. The experimental conditions in the greenhouse were 25±5°C, 65±5 RH, and natural day-night cycle. The experiment continued for 12 weeks. Plant growth measurements were taken at the end of the experiment. Plant height (aerial part) (cm), root length (cm), number of leaves per plant, and plant dry and fresh weights (g plant^-1^) of root and aerial parts were recorded. Plant height was taken by setting a ruler from the center of growing medium level to the tip of the long leaf of the plant and leaves were counted for each plant.

### Reisolation of fungus

**Figure-2:**
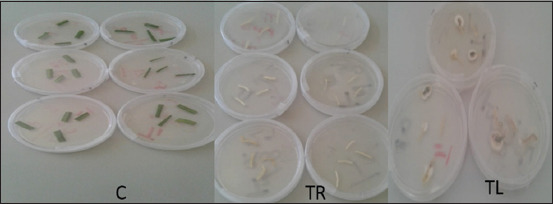
Endophytic colonization of *Tulbaghia violacea* by *Beauveria bassiana*; C: Leaf sections from control-treated plants showing no fungal outgrowth, while fungal hyphae outgrowth occurred on root (TR) and leaf (TL) sections of fungus-treated plants.

Reisolation of *B. bassiana* in tissue of *T. violacea* was determined at 3 weeks following the inoculation with *B. bassiana*. Randomly selected seedlings were carefully removed from pots and the leaves separated from the roots. The leaves and roots were then softly washed with tap water and then placed on sterile tissue paper in a laminar flow cabinet. From these, four leaf (1-2 mm^2^) and root (1 cm long) sections were excised. These parts were sterilized with 70% ethanol for 1 min, 1% sodium hypochlorite for 1 min, rinsed twice with sterilized distilled water, and placed separately on the surface of the selective medium; half-strength PDA (19.5 g/L) amended with 0.02 g/L of ampicillin (Sigma-Aldrich) and 0.04 g/L streptomycin (Sigma-Aldrich), maintained at 25°C. The presence and absence of *B. bassiana* growth on the section were recorded after 10 days. A total of 60 plants were examined in test and control treatments. The presence of *B. bassiana* in at least one of the leaf sections was considered as an indication of successful colonization of a plant (Figure-2). The data were expressed as percentage colonization ([number of plant replicates colonized/ number of plant replicates excised]×100).

### Plant material and extract preparation

Extracts of *T. violacea* were prepared by manually crushing 10 g of fresh leaves and roots separately using a porcelain mortar for 15 min followed by extraction with 25 ml of acetone. The extraction process lasted 5 h followed by filtration with Whatman filter paper no. 1 into clean centrifuge tube. Thereafter, a two-fold serial dilution was carried out; 5 ml of the filtrate yielded was mixed with 5 ml of pure acetone to obtain the 20 w/v% extract solution and 10 w/v% was obtained by taking out 5 ml from the 20 w/v% and mixed with 5 ml of clean acetone. Finally, 5 ml was taken from the 10 w/v% of extract and mixed with 5 ml of pure acetone to obtain the 5 w/v% of extract.

### Headspace gas chromatography–mass spectrometry (GC–MS) analysis

#### Sample preparation

Roots and leaves were cut off fresh *T. violacea* plants and frozen at −80°C (overnight). The leaf and root samples were freeze-dried and liquid nitrogen (N_2_) was added. The samples were immediately crushed and 1 g was weighed into a solid-phase microextraction (SPME) vial. Two milliliters of 12% alcohol solution (v/v) at pH 3.5 were added into the vial followed by 3 ml of 20% NaCl solution. The samples were vortexed and analyzed by SPME-GC-MS (with a gray fiber (divinylbenzene/carboxen/polydimethylsiloxane [DVB/CAR/PDMS]). About 5 ml of Milli-Q (ultra-pure) water was added to 5 ml of the sample into a SPME vial followed by addition of 3 ml of 20% sodium chloride (NaCl) solution and vortexed. The headspace of the sample was analyzed using a DVB/CAR/PDMS SPME fiber (gray).

#### Chromatographic separation

Chromatographic separation was carried out on a GC (6890N, Agilent Technologies Network) coupled to an Agilent Technologies Inert XL electron ionization/chemical ionization mass selective detector (5975B, Agilent Technologies Inc., Palo Alto, CA). The carrier gas was helium, and it was used at a flow rate of 1 ml/min. The following conditions were maintained: injector temperature 250°C; the split ratio 5:1; the oven temperature was set to 35°C for 6 min, at a rate of 3°C/min to 70°C for 5 min, then at 4°C/min to 120°C for 1 min, and finally increased to 240°C at a rate of 20°C/min for 2.9 min. The electron impact mode of the mass spectrometer was maintained at ionization energy of 70 eV, scanning from 35 to 500 m/z. The identification of the volatile compounds was achieved through mass spectrum and retention time comparison at 90% matching with internal standards and reference library.

#### Repellency bioassay

**Figure-3:**
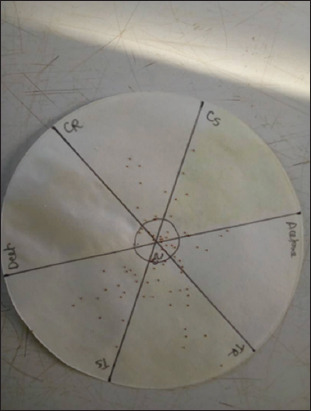
Disk repellency bioassay of *Tulbaghia violacea* on *Rhipicephalus appendiculatus* larvae. The Whatman No.1 filter paper was divided into six sections, representing treatments (TR [extracts of roots from plants treated with *B. bassiana*], TL [extracts of leaves from plants treated with *B. bassiana*], CR [extracts of roots from plants not exposed to the fungus], CL [extracts of leaves from plants not exposed to the fungus], DEET, and acetone).

A disk bioassay was used in the repellency bioassay. A 12.5 cm diameter Whatman filter paper was divided into six sections of similar dimension by drawing diametric lines passing through the center of the filter paper with a pencil and a small circle in the middle, which served as a neutral tick release zone. Each of the six sections represented a treatment: TR (extracts of roots from plants treated with *B. bassiana*), TL (extracts of leaves from plants treated with *B. bassiana*), CR (extracts of roots from plants not exposed to the fungus), CL (extracts of leaves from plants not exposed to the fungus), N,N-Diethyl-m-toluamide, and acetone (Figure-3). A small circular area (2 cm diameter) was drawn at the center of the filter paper (neutral) for the release of ticks. This test was made-up of three concentrations of plant extracts (20, 10, and 5% w/v). Each concentration had five replicates. One hundred 14-day-old larvae of *R. appendiculatus* were released on the neutral section of the treated filter papers using a fine painter’s brush (no. 3). The positions of ticks on each section were recorded 3 min after their release. Sections with the lowest number of ticks were considered to be repellent.

### Statistical analysis

The experimental data collected, mean difference in plant growth parameters, mean percentage, mean percentage of repellent bioassay, and mean area ratios of volatile compounds were analyzed using one-way analysis of variance and Tukey’s honest significant difference test was used to separate the means at a level of significance, p<0.05. These computations were performed using PAST, version 3.21 [41].

## Results

### Reisolation of fungus from leaf and root materials

*B. bassiana* successfully colonized leaf and root tissues of *T. violacea* and was reisolated from the leaf and root samples of fungus-exposed plants. The growth of fungal hyphae out of the leaf and root sections occurred in 75% and 91.6% of plants, respectively.

### Growth parameters

Plant height (aerial plant) of *T. violacea* varied significantly (df=1, 98; f=8.5; p<0.05) between the fungus and control treatments. A higher mean plant height (34.1±0.4 cm) was obtained in the fungus-exposed plants (Table-1) compared to the control (32.1±0.6 cm). Correspondingly, a longer root length (25.3±0.5 cm) was also obtained in fungus-treated plants (df=1, 98; f=4.4; p<0.05). However, the mean number of leaves per plant was not statistical different between fungus-treated plants and those in control treatment (df=1, 98; f=1.86; p>0.05); it is worth noting that a higher leaf number was obtained in the control (22.7±1.0 cm). Other growth parameters, such as dry aerial and root weights of *T. violacea*, did not significantly vary between treatments, 12 weeks post-treatment (df=1, 48; p>0.05) (Table-1). There was no significant difference in fresh shoot weight of *T. violacea* between the two treatments at 12 weeks post-treatment (df=1, 98; F=0.01; p>0.05). As well, the fresh root weight showed no significant difference (df=1, 98; f=0.86; p>0.05) (Table-1) between the two treatments.

**Table-1:** The effects of *Beauveria bassiana* inoculation on different growth parameters (mean±SE) of *Tulbaghia violacea* at 12 weeks post-treatment.

Parameters	Treatment	Period	Mean±SE
Plant height (cm)	Fungus	Week 12	34.1±0.37a
	Control	Week 12	32.1±0.56b
Root length (cm)	Fungus	Week 12	25.3±0.54a
	Control	Week 12	23.5±0.63b
Number of leaves per plant	Fungus	Week 12	20.7±1.06a
	Control	Week 12	22.7±1.00a
Leaf fresh weight (g plant^-1^)	Fungus	Week 12	17.2±0.64b
	Control	Week 12	20.4±0.54a
Root fresh weight (g plant^-1^)	Fungus	Week 12	24.2±0.84a
	Control	Week 12	23.0±0.97a
Leaf dry weight (g plant^-1^)	Fungus	Week 12	3.1±0.28a
	Control	Week 12	3.3±0.23a
Root dry weight (g plant^-1^)	Fungus	Week 12	3.1± 0.17a
	Control	Week 12	2.9± 0.24a

Means followed by lowercase letters in the same column are significantly different (p<0.05) for each parameter following comparison using Tukey’s test. Gray and white colors are used to differentiate/separate the growth parameters

### Volatile compounds

In the present study, a variety of compounds was detected following GC–MS analysis (Table-2). Some well-known anti-tick/insect compounds, such as dimethyl disulfide (DMDS), alpha-pinene, limonene, 4-methyl-2-pentanol, cumene, gamma-terpinene, and naphthalene, were detected from plants in both treatments. In general, the diversity of the compounds obtained in the fungus-exposed and unexposed plants was statistically similar (p>0.05). However, five significant variations (df=1, 4; p<0.05) in quantity of the detected compounds (ratio of area of chromatogram of a compound in relation to its corresponding internal standard) between fungus-inoculated and -control plants were observed (Table-2).

**Table-2:** Mean area ratio (in relation to internal standard) of volatile compound (mean±SE) of aerial (leaf) and root parts of *Tulbaghia violacea* following exposure to control and fungus treatments.

Volatile compounds	Leaves		Roots	
	
	*Beauveria bassiana* treatment	Control treatment	*Beauveria bassiana* treatment	Control treatment
Dimethyl disulfide	0.5±0.24a	0.2±0.09a	1.5±0.18a	0.6±0.30a
Alpha-pinene	0.1± 0.02a	0.1± 0.03a	0.1±0.02 a	-
Limonene	2.4±0.53a	2.5±0.65a	1.9±0.65a	2.3±0.53a
Cumene	0.1±0.00a	0.1±0.0a	0.1±0.0a	0.1±0.15a
Gamma-terpinene	0.5±0.02a	0.4±0.10a	0.6 ±0.01a	0.3±0.11a
Styrene	1.9±0.33a	1.2±0.37a	4.6±0.58b	26±0.77a
Octenone	0.1±0.0a	0.1±0.00a	0.4±0.33a	0.1±0.00a
Dimethyl trisulfide	0.3±0.07a	0.4±0.26a	1.3 ±0.3a	0.9±0.63a
Nonanal	0.29±0.03a	3.8±1.72b	-	4.0±1.5a
Sec-butyl sultany	0.2±0.00a	0.1±0.03b	0.2±0.01a	1±0.04a
Isodurene	0.3±0.17a	0.6±0.39a	0.2±0.05a	0.3±0.23a
(1,1-dimethylpropyl) benzene	0.08±0.00a	0.08±0.03a	0.1±0.02a	0.09±0.01a
Isodurene (trans)	1.2±0.09b	0.8±0.02a	0.2±0.04b	0.8±1.62a
4-methyl-indene	0.3±0.02a	0.2±0.08a	0.5±0.01a	0.3±0.10a
2-2-Sulfanyl ethyl	0.3±0.04a	0.2±0.09a	1.5±0.18a	0.7±0.30a
Alpha-cyclocitral	0.12±0.03a	-	-	-
Methyl (methylthio) methyl	4.4±1.2a	1.8±0.90a	25.1±4.58a	9.9±6.7 b
Trans(+)-carveol	-	0.3±0.32a	4.0±1.37a	1.3±0.70a
Naphthalene	0.9±0.09a	0.6± 0.1b	1.7±0.2a	1.2±0.40b
1,2,4-trithiolane	0.1±0.02a	0.2±0.13a	0.1±0.06a	0.1± 0.10a
Cis-genenaineol	0.3±0.01a	0.1±0.03a	0.2±0.00a	0.1±0.01a
Dimethyl trithiocarbonate	0.1±0.03a	0.1±0.03a	-	0.1±0.03a
4-Propylbenzamide	0.2±0.16a	0.1±0.03a	0.7±0.13a	0.2±0.05a
Tris(methylthio met)	0.2±0.05a	0.1±0.03a	0.7±0.01a	0.5±0.01a
9,12-octadecadienoic-acid-ethyl-ester	-	-	0.05 ±0.00a	-

Means followed by same lowercase letters in the same row are not significantly different (p>0.05) following comparison of fungus and control treatments using Tukey test; roots and leaves were analyzed separately

### Repellency bioassay

When repellent activities between test and control extracts of *T. violacea* were compared at the highest concentration, significantly (df=5, 24, f=8.7; p<0.01) more larvae (20.4±4.3-21.4±2.0) of *R. appendiculatus* were found in the section treated with extracts from the plants exposed to the fungal inoculum compared to the control (Table-3). In general, significantly (df=5, 24; p<0.01) increased tick repellency was observed at the lower concentrations (10 and 5 w/v%) compared to the highest concentration. The results further indicated that the root extracts of plants exposed to extracts from fungus-exposed plants performed better than the corresponding control extracts in the repellency bioassay at the lower concentrations (10 and 5 w/v%) (Table-3).

**Table-3:** The mean±SE number of *Rhipicephalus appendiculatus* larvae that were found on the fungus and control treated sections after 3 min after exposure to varying concentrations of acetone extracts in a disk repellency bioassay.

Treatments	Mean number of ticks present±SE 20% w/v	Mean number of ticks present±SE 10% w/v	Mean number of ticks present±SE 5% w/v
Test (fungus) leaves (TL)	21.4±2.0bA	8.4±2.8abB	7.8±2.1aB
Test (fungus) roots (TR)	20.4±4.3bA	5.6±1.9aB	6.8±2.7aB
Control leaves (CL)	5.0±1.2aA	9.0±2.6abA	8.2±2.5aA
Control roots (CR)	9.6±2.0aB	17.2±4.1bA	14.2±1.4aB
DEET	6.0±1.9aA	2.8±1.0 aB	3.8±1.3 aB
Acetone	9.4±2.1aA	11.0±2.0 abA	10.8±2.8 aA

Means followed with the same lowercase letters on the same column are not significantly different, following Tukey’s test at p>0.05 level of significance. Means followed with the same uppercase letters on the same row are not significantly different, following Tukey’s test at p>0.05 level of significance

## Discussion

The fungal strain of *B. bassiana* used in this study was successfully reisolated from the leaf and root tissues, suggesting that the fungus colonized the tissues of *T. violacea*. Successful colonization of many plant species following inoculation with *B. bassiana* has been reported previously [42-44]. Furthermore, this study demonstrated that soil drench inoculation with conidia of entomopathogenic fungi *B. bassiana* had variable effects on the different growth parameters – *B. bassiana* did not significantly influence the number of leaves, and dry and fresh weights of *T. violacea* when compared with the control plants, but it did influence plant height and root length significantly (p<0.05). A recent review by Akello *et al*. [28] and Dara *et al*. [45] indicated that *B. bassiana* showed positive influence on the survival, growth, length, and dry weight of cabbage. However, Jaber and Enkerli [46] argued that there is an absence of consistency in the plant growth promotion obtained by inoculating plants with entomopathogenic fungi.

In this study, phytochemical analysis revealed that the volatile constituents in the extract of *T. violacea* varied between control and fungus treatments. Some of the compounds contained in the leaf and root extracts of *T. violacea* evaluated in this study were also reported by Olorunnisola *et al*. [47]. Interestingly, sulfur-based compounds, such as DMDS, are among compounds that were also detected in the current study even though it was not significantly different between treatments. These compounds are released by quite a number of plants to the environment, and they are toxic to some insects; Dugravot *et al*. [48] demonstrated that Dimethyl disulfide (DMDS) can induce an uncommon complex neurotoxic activity. Geraniol is commonly used as an insecticide and it exhibits various biochemical and pharmacological properties [49]. A prominent anti-insect compound that was detected in this study is naphthalene [49]; it is the main anti-insect active ingredient in mothballs.

Even though arthropod-pathogenic fungi and plant extracts have been evaluated with promising results against tick species, this is the first study that evaluated the effects of inoculating plants with fungal inoculum and its subsequent effects on anti-tick activity of plant extracts. The use of EPF is one of the approaches being considered as an alternative to chemical acaricides [50]. In the present study, the acetone extracts of *T. violacea* that were inoculated with *B. bassiana* inoculum were assessed against larvae of *R. appendiculatus*. There was a significant difference in tick repellency among extracts of *T. violacea* that were inoculated with fungi when compared with the control treatment. At a higher concentration of 20 w/v% concentration, extracts from fungus-inoculated plants performed poorly, even showing a relative net positive attraction. However, root extracts of fungus-treated *T. violacea* repelled more ticks at lower concentrations (5 w/v% and 10 w/v%) than the corresponding control. Variations in the concentrations of specific bioactive compounds might have influenced the results observed in this study. Previously, Nchu *et al*. [51] reported that dichloromethane extract of garlic showed positive repellent effects on ticks at a lower range of concentrations in repellency bioassays. In a review paper, Wanzala *et al*. [52] presented tick repellent activities of essential oils from different plant species.

The development of efficient and sustainable agro-technologies for the production of high-quality medicinal materials with enhanced therapeutic properties is needed to meet the demands of the pharmaceutical industry, traditional healers, and the cosmetics industry [31,53]. Medicinal plant cultivation would help farmers to generate important monetary returns, help conserve medicinal plants in the wild and help preserve traditional ethnomedicinal knowledge [54].

## Conclusion

Broadly, the inoculation of *T. violacea* with an endophytic arthropod-pathogenic fungus influenced the secondary metabolite contents as well as the tick repellency of the plant extracts of *T. violacea*. These findings contribute toward a better understanding of the role of fungal endophytes in influencing secondary metabolite production and bioactivity of plant extracts and open up the possibility of developing innovative cultivation approaches for medicinal plants.

## Authors’ Contributions

PS, NN, GM, YPN, and FN conceptualized and designed this research. The research was carried out by PS and YPN. FN and PS analyzed the data and result. PS drafted the first version of the manuscript. PS, NN, GM, YPN, and FN revised and finalized the manuscript. All authors read and approved the final manuscript.

## References

[1] Nyahangare E.T, Mvumi B, Mutibvu T (2015). Ethnoveterinary plants and practices used for ecto-parasite control in semi-arid smallholder farming areas of Zimbabwe. J. Ethnobiol. Ethnomed.

[2] Jongejan F, Uilenberg G (2004). The global importance of ticks. Parasitology.

[3] Jabbar A, Abbas T, Saddiqi H.A, Qamar M.F, Gasser R.B (2015). Tick-borne diseases of bovines in Pakistan:Major scope for future research and improved control. Parasit. Vectors.

[4] Minjauw B, McLeod A (2003). Tick-Borne Diseases and Poverty:The Impact of Ticks and Tick-Borne Diseases on the Livelihoods of Small-Scale and Marginal Livestock Owners in India and Eastern and Southern Africa. DFID Animal Health Program.

[5] Norval R.A.I, Perry B.D, Young A.S (1992). The Epidemiology of Theileriosis in Africa.

[6] Hoogstraal H (1985). Argasid and nuttalliellid-ticks as parasites and vectors. Adv. Parasitol.

[7] Uilenberg G (1999). Immunization against diseases caused by *Theileria parva*:A review. Trop. Med. Int. Health.

[8] Gachohi J, Skilton R, Hansen F, Ngumi P, Kitala P (2012). Epidemiology of East Coast fever (*Theileria parva* infection) in Kenya:Past, present and the future. Parasit. Vectors.

[9] Smith E.R, Parker D.M (2010). Tick communities at the expanding wildlife/cattle interface in the Eastern Cape Province, South Africa:Implications for corridor disease. J. S. Afr. Vet. Assoc.

[10] Rajput Z.I, Hu S.H, Chen W.J, Arijo A.G, Xiao C.W (2006). Importance of ticks and their chemical and immunological control in livestock. J. Zhejiang Univ. Sci. B.

[11] Adenubi O.T, Fasina F.O, McGaw L.J, Eloff J.N, Naidoo V (2016). Plant extracts to control ticks of veterinary and medical importance:A review. S Afr. J. Bot.

[12] Magwede K, Tshisikhawe M.P, Luseba D, Bhat R.B (2014). Ethnobotanical survey of medicinal plants used in treatment of ticks. Int. J. Exp. Bot.

[13] Masika P.J, Sonandi A, Van Averbeke W (1997). Perceived causes, diagnosis and treatment of babesiosis and anaplasmosis in cattle by livestock farmers in communal areas of the Central Eastern Cape Province, South Africa. J. S. Afr. Vet. Assoc.

[14] Van Wyk B.E, Van Oudtshoorn B, Gericke N (1997). Medicinal Plants of South Africa.

[15] Eloff J.N (1998). Which extractant should be used for the screening and isolation of antimicrobial components from plants?. J. Ethnopharmacol.

[16] Newman D.J, Cragg G.M (2007). Natural products as sources of new drugs over the last 25 years. J. Nat. Prod.

[17] Maia M.F, Moore S.J (2011). Plant-based insect repellents:A review of their efficacy, development and testing. Malar. J.

[18] Khare R.K, Das G, Kumar S, Bendigeri S, Sachan S, Saiyam R, Banerjee D.K, Khare D.S (2019). Herbal insecticides and acaricides:Challenges and constraints. Int. J. Chem. Stud.

[19] Yang L, Wen K.S, Ruan X, Zhao Y.X, Wei F, Wang Q (2018). Response of plant secondary metabolites to environmental factors. Molecules.

[20] Coley P.D (1987). Interspecific variation in plant anti-herbivore properties:The role of habitat quality and rate of disturbance. New Phytol.

[21] Ncube B, Finnie J.F, Van Staden J (2012). Quality from the field:The impact of environmental factors as quality determinants in medicinal plants. S. Afr. J. Bot.

[22] Adolfsson L, Nziengui H, Abreu I.N, Šimura J, Beebo A, Herdean A, Aboalizadeh J, Široká J, Moritz T, Novák O, Ljung K, Schoefs B, Spetea C (2017). Enhanced secondary-and hormone metabolism in leaves of arbuscular mycorrhizal *Medicago truncatula*. Plant Physiol.

[23] Ding C.H, Wang Q.B, Guo S, Wang Z.Y (2018). The improvement of bioactive secondary metabolites accumulation in *Rumex gmelini* Turcz through co-culture with endophytic fungi. Braz. J. Microbiol.

[24] Zhai X, Chen L, Jia M, Li C.H, Shen H, Ye B.Z, Qin L.P, Han T (2017). A Stable Beneficial Symbiotic Relationship between Endophytic Fungus S *chizophyllum**commune* and Host Plant *Panax ginseng*.

[25] Bamisile B, Dash C.K, Akutse K.S, Keppanan R, Wang L (2018). Fungal endophytes:Beyond herbivore management. Front. Microbiol.

[26] Parsa S, Ortiz V, Vega F.E (2013). Establishing fungal entomopathogens as endophytes:Towards endophytic biological control. J. Vis. Exp.

[27] Parisi P.A.G, Grimoldi A, Omacini M (2014). Endophytic fungi of grasses protect other plants from aphid herbivory. Fungal Ecol.

[28] Akello J, Dubois T, Gold C.S, Coyne D, Nakavuma J, Paparu P (2007). *Beauveria bassiana*(Balsamo) Vuillemin as an endophyte in tissue culture banana (*Musa*spp.). J. Invertebr. Pathol.

[29] Muvea A.M, Meyhöfer R, Subramanian S, Poehling H.M, Ekesi S, Maniania N.K (2014). Colonization of onions by endophytic fungi and their impacts on the biology of *Thrips tabaci*. PLoS One.

[30] Jia M, Chen L, Xin H.L, Zheng C.J, Rahman K, Han T, Qin L.P (2016). A friendly relationship between endophytic fungi and medicinal plants:A systematic review. Front. Microbiol.

[31] Nchu F, Matanzima Y, Laubscher C.P, Amanullah K, Fahad S (2018). Prospects of N fertilization in medicinal plant cultivation. Nitrogen in Agriculture-Updates.

[32] Jarial M.S (2001). Toxic effect of garlic extracts on the eggs of *Aedes aegypti*(*Diptera*
*Culicidae*):A scanning electron microscopic study. J. Med. Entomol.

[33] Nchu F, Magano S.R, Eloff J.N (2005). *In vitro* investigation of the toxic effects of extracts of *Allium sativum* bulbs on adults *of Hyalomma marginatum rufipes* and *Rhipicephalus pulchellus*. J. S. Afr. Vet. Assoc.

[34] Borlinghaus J, Albrecht F, Gruhlke M.C, Nwachukwu I.D, Slusarenko A.J (2014). Allicin:Chemistry and biological properties. Molecules.

[35] Lyantagaye S.L (2011). Ethnopharmacological and phytochemical review of *Allium* species (sweet garlic) and *Tulbaghia* species (wild garlic) from Southern Africa. Tanzan. J. Sci.

[36] Harris S (2004). Tulbaghia violacea. Free State National Botanical Garden.

[37] Mander M, McKenzie M (2005). Southern African trade directory of indigenous natural products. Commercial Products from the Wild Group.

[38] Van Wyk A.B, Oudtshoorn B, Gericke N (2009). South African Medicinal plants, South Africa.

[39] Netshiluvhi T.R, Eloff J.N (2016). Effect of water stress on antimicrobial activity of selected medicinal plant species. S. Afr. J. Bot.

[40] Inglis G.D, Enkerli J, Goettel M.S, Lacey L.A (2012). Laboratory techniques used for entomopathogenic fungi *Hypocreales*. Manual of Techniques in Invertebrate Pathology.

[41] Hammer Ø, Harper D.A.T, Ryan P.D (2001). PAST:Paleontological statistics software package for education and data analysis. Palaeontol. Electron.

[42] Tefera T, Vidal S (2009). Effect of inoculation method and plant growth medium on endophytic colonization of sorghum by the entomopathogenic fungus *Beauveria bassiana*. Biocontrol.

[43] Sánchez-Rodríguez A.R, Antonio Rafael Sánchez-Rodríguez Del Campillo M.C, Quesada-Moraga E (2015). *Beauveria bassiana*:An entomopathogenic fungus alleviates Fe chlorosis symptoms in plants grown on calcareous substrates. Sci. Hortic.

[44] Waqas M, Khan A.L, Hamayun M, Shahzad R, Kang S.M, Kim J.G, Lee I.J (2015). Endophytic fungi promote plant growth and mitigate the adverse effects of stem rot:An example of *Penicillium citrinum* and *Aspergillus terreus*. J. Plant Interact.

[45] Dara S.K (2017). Impact of entomopathogenic fungi on the growth, development, and Health of cabbage growing under water stress. Am. J. Plant Sci.

[46] Jaber L.R, Enkerli J (2017). Fungal entomopathogens as endophytes:Can they promote plant growth?. Biocontrol Sci. Technol.

[47] Olorunnisola O.S, Bradley G, Afolayan A. J (2012). Chemical composition, antioxidant activity and toxicity evaluation of essential oil of *Tulbaghia violacea* Harv. J. Med. Plants Res.

[48] Dugravot S, Grolleau F, Macherel D, Rochetaing A, Hue B, Stankiewicz M, Huignard J, Lapied B (2003). Dimethyl disulfide exerts insecticidal neurotoxicity through mitochondrial dysfunction and activation of insect KATP channels. J. Neurophysiol.

[49] Daisy B.H, Strobel G.A, Castillo U, Ezra D, Sears J, Weaver D.K, Runyon J.B (2002). Naphthalene, an insect repellent, is produced by *Muscodor vitigenus* a novel endophytic fungus. Microbiology.

[50] Erler F, Ates A.O (2015). Potential of two entomopathogenic fungi *Beauveria bassiana* and *Metarhizium anisopliae*(*Coleoptera*:Scarabaeidae), as biological control agents against the June beetle. J. Insect Sci.

[51] Nchu F, Magano S.R, Eloff J.N (2016). Repellent activities of dichloromethane extract of *Allium sativum*(Garlic) (*Liliaceae*) against *Hyalomma rufipes*(*Acari*). J. S. Afr. Vet. Assoc.

[52] Wanzala W, Hassanali A, Mukabana W.R, Takken W (2014). Repellent activities of essential oils of some plants used traditionally to control the brown ear tick *Rhipicephalus appendiculatus*. J. Parasitol. Res.

[53] Bourgaud F, Gravot A, Milesi S, Gontier E (2001). Production of plant secondary metabolities:A historical perspective. Plant Sci.

[54] Silori C.S, Badola R (2000). Medicinal plant cultivation and sustainable development. Mt. Res. Dev.

